# *In vitro* test to evaluate survival in the gastrointestinal tract of commercial probiotics

**DOI:** 10.1016/j.crfs.2021.04.006

**Published:** 2021-05-12

**Authors:** Maritiele Naissinger da Silva, Bruna Lago Tagliapietra, Vinícius do Amaral Flores, Neila Silvia Pereira dos Santos Richards

**Affiliations:** a Universidade Federal de Santa Maria, Departamento de Tecnologia e Ciência de Alimentos, Rua Antonio Botega, 270, CEP 97095-030, Santa Maria, RS, Brazil; b Universidade Estadual de Campinas, Departamento de Tecnologia de Alimentos, Campinas, SP, Brazil; c Universidade Federal de Santa Maria, Departamento de Tecnologia e Ciência Dos Alimentos, Santa Maria, RS, Brazil

**Keywords:** Functional foods, Gastrointestinal simulation, Symbiotic, Supplements, Viability

## Abstract

The search for functional foods grows constantly, and in this demand, the supply of industries that seek to produce and sell supplements also grows, as is the case of probiotics freely sold in pharmacies and supermarkets. Given a large number of foods with probiotic appeal and supplements sold without the need for a nutritional or medical prescription, this study came up to evaluate the viability of commercial probiotic cells, through in vitro gastrointestinal simulation and analyzing the information present in their labeling. Eleven commercial probiotic samples were analyzed, and viable cell counts were performed before and after in vitro simulation. These products usually use appealing labeling and induce the consumer to purchase these probiotics, which often do not offer the benefits described on the packaging. The results showed that only two samples had the initial concentration indicated on their labeling and four samples offered a concentration of 3 log CFU g^−1^ in the ileum portion. All samples had a reduction in concentration during the gastrointestinal simulation, which varied from 1 to 4 log CFU g^−1^, but most do not fulfill the offer of a probiotic supplement, and there should be more inspection and control over the commercialization of this product niche.

## Highlights

• Of eleven probiotics analyzed, only two were in accordance with their labeling.
• Only six probiotics showed an initial concentration above 8 log CFU g-1.
• After gastrointestinal simulation, six probiotics showed viability greater than 6 log CFU g-1.
• Probiotic fermented milk and microorganisms protected by capsules showed the best results.
• Technologies are needed that contribute to maintaining probiotic viability in storage and digestion.

## Graphical abstract

**Figure:**
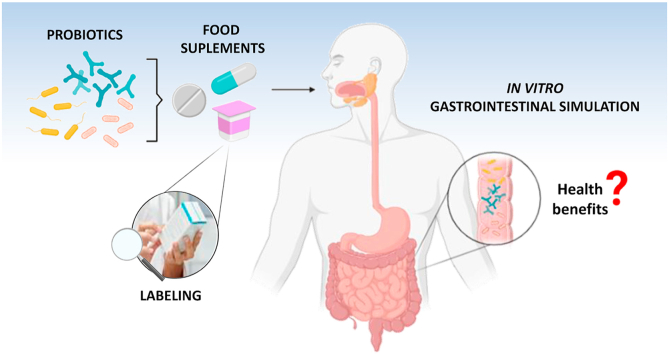

## 1 Introduction

The search for functional foods has increased considerably in the last decades since foods have come to be seen not only as a source of nutrients but also as promoters of health and well-being (Rouxinol-dias et al., 2016; Misra et al., 2021). As a result, there is growing interest from industries in the development of probiotic dietary supplements that promote health benefits.

Supplements are products for oral ingestion, presented in pharmaceutical forms, intended to supplement the diet of healthy individuals with nutrients, bioactive substances, enzymes, or probiotics, isolated or combined (Brazil, 2018a) and can be ingested in different ways, such as powder, capsules, and tablets (Caillard and Lapointe, 2017).

Probiotics can be defined as live microorganisms that, when administered in adequate amounts, confer a benefit on the health of the host (FAO/WHO, 2006; Hill et al., 2014; Brazil, 2018a). These microorganisms contribute directly or indirectly to several health benefits, including protection against pathogenic microorganisms, hypertension, inflammation, diabetes, oxidative stress, and are also involved in the modulation of the microbiome, immune modulation, and anti-cholesterolemic activity (Novik and Savich, 2020). To promote their beneficial effects on the host, probiotics must survive gastrointestinal transit, the acidic conditions of the gastric environment and be able to reach the large intestine in adequate amounts to allow colonization and proliferation.

Probiotics help to restore the intestinal microbiota, through the adhesion and colonization of the intestinal mucosa, preventing the proliferation of pathogenic bacteria, in addition to competing for nutrients available in the ecological niche (Saad, 2006). Symbiotics, on the other hand, can be defined as a mixture of probiotics and prebiotics that beneficially affect the host, selectively stimulating growth and/or activating the metabolism of health-promoting bacteria, thus improving the host's well-being (Ouwehand et al., 2007).

With the property of adhering to the intestinal epithelial cells, probiotics can improve the microbiota and the digestive process, protect against pathogens and generate potential anticarcinogenic properties (Verruck et al., 2020). However, most probiotics cannot survive in large quantities due to the low pH of gastric juice, which limits its effectiveness in most functional foods (Shori, 2017). Resistance to stomach acid and tolerance to bile salts are two fundamental properties for microorganisms to be considered probiotics, allowing them to survive acidic stomach conditions and the presence of bile salts in the small intestine during passage through the gastrointestinal tract (Carvalho Lima et al., 2009; Saad, 2006).

For a food to be marketed with claims to bring health benefits, due to the addition of probiotics, it must contain viable cells from probiotic cultures of at least 10^6^–10^7^ ​CFU/g or in the portion to be consumed (FAO/WHO, 2006; Hill et al., 2014), and for beneficial action to occur in the intestine, they must be able to survive processing and storage conditions, be ingested in adequate quantities, reaching the viable number of microorganisms (Ranadheera et al., 2019; Rasika et al., 2020).

Probiotics should be present in food in an amount of 8–9 log CFU g^−1^ in the daily recommendation of the product (Vasiljevic and Shah, 2008), before ingestion, to ensure that a sufficient therapeutic minimum of 6–7 ​CFU ​g^−1^ can reach the colon (Nazzaro et al., 2009; Hill et al., 2014).

The main difficulty encountered by industries in adding probiotic bacteria to functional foods is related to the maintenance and viability of these cultures. In vitro digestion models are widely used to study, through the simulation of gastrointestinal conditions, structural changes, digestibility, and the release of compounds present in food (Hur et al., 2011; Jacobsen et al., 2020).

Proof of benefit for probiotics requires proof of survival to conditions in the human digestive tract and evidence of the effect on humans obtained through studies that are conducted with the lineage of the microorganism; that involves a representative group of the population of interest or whose results can be extrapolated to that of interest; that consider the minimum amount suggested to obtain the benefit; to assess outcomes relevant to the claimed benefit; and minimize bias and factors that can confuse the consumer (Caillard and Lapointe, 2017, Hill et al., 2014; Brazil, 2018a, 2019).

Thus, several researchers have already evaluated the resistance of microorganisms in gastrointestinal fluids, simulating the digestive process by in vitro tests (Fávaro-Trindade and Grosso, 2002, Liserre et al., 2007; Gbassi et al., 2009; Gebara et al., 2013; Caillard and Laiponte, 2017; Jin et al., 2019; Prestes et al., 2021). Studies with oral probiotic supplements are incipient, therefore, the objective of this study was to evaluate the viability, through gastrointestinal simulation in vitro, of probiotics marketed as dietary supplements compared with the information available on the product label.

## 2 Material and methods

### 2.1 Commercial probiotics

Eleven commercial probiotics were analyzed and given the name according to Table 1. Of these, three were supplied by specialized companies and can be used for product development (CP1, CP2, and CP3). The MP4, MP5, and MP11 samples were purchased from handling pharmacies, with no indication of the method of consumption and preparation. The samples CP6, CP7, CP8, and CP9 were obtained as probiotic supplements in conventional pharmacies, and the sample FM10, which is probiotic fermented milk, was acquired in a supermarket. The in vitro experiment was carried out in triplicate with samples from different manufacturing batches and within the expiration date. The storage conditions indicated on the label of each product were maintained.

**Table 1:** Commercial probiotics are used to assess viability during the gastrointestinal simulation.

Sample	Strain^a^	Concentration^a^	Presentation^a^	Storage indication^a^	Preparation indication^a^	Indication of consumption^a^	Shelf life^a^
IP1	*L. rhamnosus*	NE	Lyophilized	5 ​± ​3 ​°C	Activation	NE	12 months
IP2	*L. acidophilus* LA-14	NE	Lyophilized	5 ​± ​3 ​°C	Activation	NE	12 months
IP3	*L. acidophilus* LA-14	NE	Lyophilized	4 ​°C	Activation	NE	12 months
MP4	*L. acidophilus* LA-14	NE	Lyophilized	Refrigeration	Dilute with water	NE	NE
MP5	*L. acidophilus* LA-14	NE	Lyophilized	Refrigeration	Dilute with water	NE	NE
CP6	*L. acidophilus* LA 14	9 log CFU g^−1^	Capsule	Ambient temperature	Ready for consumption	1 capsule (916 ​mg)/day	NE
CP7	*L. acidophilus* NCFM	10 log CFU g^−1^	Capsule	Ambient temperature	Ready for consumption	1 to 2 capsule (335 ​mg)/day	24 months
	*L. paracasei* Lpc-37						
	*B. lactis* Bi-07						
	*B. lactis* BI-04						
	*B. bifidum* Bb-02						
CP8	Frutooligossacarídeo	5,5 ​g	Lyophilized	Ambient temperature	Dilute with water	1 sachet (7g)/day	24 months
	*L. acidophilus* SD 5221	9 log CFU g^−1^					
	*L. rhamnosus* SD 5217	9 log CFU g^−1^					
	*B. bifidum* SD 6576	9 log CFU g^−1^					
CP9	Frutooligossacarídeo	6,0 ​g	Lyophilized	Ambient temperature	Dilute with water	1 to 2 sachet (6–12g)/day	24 months
	*L. acidophilus* SD 5221	9 log CFU g^−1^					
	*L. rhamnosus* SD 5675	9 log CFU g^−1^					
	*L. paracasei* SD 5275	9 log CFU g^−1^					
	*B. lactis* SD 5674	9 log CFU g^−1^					
FM10	*B. animalis* CNCM I-2494	NE	Liquid	1–10 ​°C	Ready for consumption	2 shot (200 ​mL)/day	45 days
MP11	*B. bifidum* LA-14	NE	Lyophilized	Refrigeration	Dilute with water	NE	NE

a Information as described on the packaging. NE: Not Established; IP: Industrial Probiotic; MP: Manipulated Probiotic; CP: Commercial Probiotic; FM: Fermented Milk.

### 2.2 Sample preparation

Probiotic cultures (IP1, IP2, and IP3) were activated before use, as recommended by the manufacturers. The activation method used was in MRS broth (Sigma-Aldrich®), using 1 ​g of probiotic for every 100 ​mL of broth, and after incubated for 15 ​h at 37 ​°C. After the incubation for activation, the culture together with the MRS broth were centrifuged at 4670×*g* for 15 ​min in a refrigerated centrifuge, at 4 ​°C, and washed in NaCl solution (0.85% w/v) twice, thus being able to food application.

The lyophilized manipulated samples, MP4, MP5, and MP11, were subjected to the analyzes were also acquired, since there was no indication of preparation and activation before consumption. When purchased, the only recommendation received was to be kept refrigerated.

The samples acquired in conventional pharmacies, in the form of probiotic supplements, were analyzed according to their consumption indication. In samples CP6 and CP7, the probiotic strains were encapsulated, so it was the portion of a capsule for analysis. The CP8 and CP9 samples indicated consumption for dilution in water, so the dilution of 1 ​g of sample in 9 ​mL of peptone water was considered and after the analysis was carried out.

### 2.3 Counting of viable probiotic cells

The counting of viable probiotic cells was carried out in two moments: (1) after being prepared for consumption (activated, diluted, or removed from the packaging), and (2) after simulating the digestion of products, counting at each stage of the passage through the gastrointestinal tract of the in vitro experiment (esophagus/stomach, duodenum, and ileum).

To carry out the analysis, 1.0 ​g aliquots were transferred in suitable dilutions, in triplicate, to disposable Petri dishes. For the species *Lactobacillus acidophilus*, *Lactobacillus rhamnosus,* and *Lactobacillus paracasei* depth plating was performed on MRS agar (Kasvi®), and to determine the concentration of *Bifidobacterium bifidum, Bifidobacterium lactis* and *Bifidobacterium animalis,* 0.5% of solution A was added (dicloxacillin at a concentration of 0.01% w/v), 1.0% solution B (11.0% m/v lithium chloride) and 0.5% solution C (10.0% m/v cysteine chloride) on MRS agar (Kasvi®) and plating by depth. After inoculation, the plates were incubated inverted in an anaerobic jar, in a bacteriological oven at 37 ​°C for 72 ​h (Sheu and Marshall, 1993).

### 2.4 *In vitro* survival test for gastrointestinal conditions

The purpose of the gastrointestinal simulation is to know the survival and concentration of probiotic cells in the stomach, duodenum, and ileum under the presence of enzymes and pH changes, and the adherence and physiological action of samples containing probiotics were not evaluated in the present study.

**Fig. 1:**
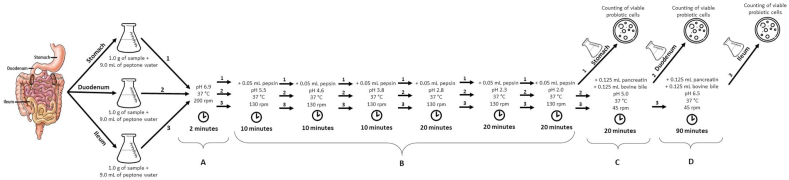
Stages of gastrointestinal simulation (esophagus/stomach, duodenum, and ileum). Addition of enzymes, adjustment of pH, and control of temperature, rpm, and time in each phase of the in vitro test. Caption: A ​= ​simulated digestion in the mouth. B ​= ​simulated stomach digestion. C ​= ​simulated digestion in the duodenum. D ​= ​simulated digestion in the ileum. The stages of in vitro digestion occurred in three erlenmeyers: (1) stomach, (2) duodenum, and (3) ileum. The first phase (A) refers to the time of the sample in the simulated passage through the mouth, being submitted to the three steps (1, 2 and 3). The second phase (B) corresponds to the time of the samples in the simulated passage through the stomach. At the end of phase B, the Erlenmeyer corresponding to the stomach (1) is removed and subjected to analysis for counting viable cells at the end of phase B. The erlenmeyers corresponding to the duodenum (2) and ileum (3) follow in the simulated digestion. In phase C, the passage through the duodenum is simulated, at the end of the time, the erlenmeyer that corresponds to the duodenum (2) is removed and subjected to analysis to count viable cells. The last phase of simulated digestion (D), contemplates only the Erlenmeyer flask corresponding to the ileum (3). In the end, the Erlenmeyer 3 is submitted and subjected to analyzes for counting viable cells.

The simulation to assess the survival of commercial probiotics against gastrointestinal conditions was carried out as described by Madureira et al. (2011), with adaptations (Fig. 1). The analysis was conducted in a refrigerated Shaker incubator (TE-421, Technal, Brazil) maintained at 37 ​°C, to simulate the temperature of the human body, and mechanical agitation was used in parallel to simulate peristaltic bowel movements, with intensities similar to those achieved in the digestive tract section.

Survival was assessed sequentially in media that simulate the different sections of the gastrointestinal tract (esophagus/stomach, duodenum, and ileum). Aliquots of 1.0 ​g of sample were added with 9.0 ​mL of peptone water, being prepared equally in three Erlenmeyer, and all submitted to the same conditions for gastrointestinal simulation.

An acid solution (HCl 0.1 ​mol ​L^−1^) and a basic solution (NaHCO_3_ 0.1 ​mol ​L^−1^) were previously prepared and autoclaved to adjust the pH of the samples throughout the gastrointestinal simulation. Initially, the pH was adjusted to 6.9, to simulate the acidity of the mouth, remaining in this condition for 2 ​min and proceeding to the next step.

In the esophagus-stomach stage, 25 ​mg ​mL^−1^ of pepsin (Sigma®), prepared in HCl 0.1 ​mol ​L^−1^, was used. This solution was added, in equal aliquots, throughout the gastric phase, to an amount of 0.05 ​mL ​mL^−1^, following six steps of pH/time (minutes): 5.5/10; 4.6/10; 3.8/10; 2.8/20; 2.3/20, and 2.0/20. The rotation of 130 ​rpm was maintained, adding pepsin (0.05 ​mL ​mL^−1^) and the pH adjusted using 0.1 ​mol ​L^−1^ HCl in each step (pH/time). At the end of this phase, one of the erlenmeyer was removed and the sample was immediately subjected to analysis of probiotic cell count survivors in the simulation of passage through the stomach.

In the step referring to the duodenum, at a concentration of 0.25 ​mL ​mL^−1^, a solution containing 2 ​g ​L^−1^ of pancreatin (Sigma®) and 12 ​g ​L^−1^ of bovine bile salts (Sigma®) was used, prepared in NaHCO_3_ 0.1 ​mol ​L^−1^, the pH is adjusted to 5.0 with the addition of the solution of pancreatin and bile enzymes (0.25 ​mL ​mL^−1^) and remaining for 20 ​min at 45 ​rpm. In the end, the second erlenmeyer was removed, which corresponded to the stage of the duodenum, and the sample was subjected to analysis of surviving probiotic cell counts.

The ileum step was carried out by increasing the pH to 6.5, using a 0.1 ​mol ​L^−1^ NaHCO^3^ solution, and remaining at 90 ​rpm for 90 ​min. In the end, the sample was subjected to analysis to count probiotic cells surviving after the simulated passage through the gastrointestinal tract.

The enzymes used were stored under freezing before use (- 18 ​°C). All solutions were prepared at the time of use and sterilized with a 0.22 ​μm pore membrane (Minisart, Sartorius Stedim Biotech, Germany).

### 2.5 Analysis of results

The data obtained were tabulated in Microsoft Office Excel® spreadsheets and the triplicates were averaged for each microbiological analysis and presented standard deviation.

## 3 Results and discussion

From the labeling of the probiotics analyzed (Table 1), it was possible to identify the composition and concentration of microorganisms in each product. It was also observed, on the label, if the products needed to go through an activation or reconstitution process. The only information applicable for all samples was the indication not to use the products in hot foods or to subject them to excessive heat, except for sample FM10, which was fermented milk, and consumption was indicated refrigerated and not mentioned on the packaging care with heating.

Among the 11 samples analyzed, three (IP1, IP2, IP3) are industrial probiotic strains and indicated for the manufacture of foods with probiotic potential, being marketed by specialized companies. Another three samples (MP4, MP5, and MP11) are lyophilized probiotics marketed to the general public in compounding pharmacies. CP6 and CP7 samples are labeled as functional foods. The CP8 sample is called a symbiotic functional food product, with the addition of fructooligosaccharides (FOS) in its composition. The CP9 sample does not have a specific sales denomination on its label, it just appears to be a symbiotic product, also with the addition of FOS.

CP6, CP7, CP9, and CP9 samples are sold in conventional pharmacies for the general public. The FM10 sample is classified within the dairy food group, being fermented milk, and is found for sale to the general public in supermarkets. It should be noted that none of the samples was requested a medical requisition for purchase from the points of sale, as well as information on the use and consumption of samples IP1, IP 2, IP 3, MP4, MP5, and MP11 was not made available.

In Table 1, it is also possible to analyze the values found for *Lactobacillus* sp. and *Bifidobacterium* sp. in each sample. Among the 11 samples, only two (FM10 And MP11) have *Bifidobacterium* sp. in isolation. The IP1, IP2, IP3, MP4, MP5, and CP6 samples are composed only by *Lactobacillus* sp. and CP7, CP8, and CP9 samples are composed of a combination of both probiotic genera.

The CP6 and CP7 samples showed concentrations corresponding to those indicated on the label, being 9.9 and 10.7 log CFU g^−1^, respectively. Samples IP1, IP2, IP3, and FM10 also showed satisfactory values, above 8 log CFU g^−1^, different from the results found for samples MP4, MP5, and MP11, which were less than 6 log CFU g^−1^. The CP9 sample showed results marked as satisfactory, being above 8 log CFU g^−1^ when considering the consumption of the 12 ​g portion by the individual, however, this concentration is still lower than indicated on its labeling. The CP8 sample showed a concentration above 6 log CFU g^−1^, also lower than that indicated on its packaging (Table 2). Hungri and Longo (2009) analyzed the viability of microorganisms in probiotic foods and also found counts of less than indicated on the product packaging.

**Table 2:** Probiotic viability of commercial microorganisms before and after gastrointestinal simulation. Analysis performed on 1g of sample.

Sample	Gastrointestinal simulation			
*Lactobacillus* sp.	Initial	Stomach	Duodenum	Ileum
IP1	8.2 ​± ​0.1^a^	8.0 ​± ​0.1	7.9 ​± ​0.1	7.7 ​± ​0.1
IP2	9.8 ​± ​0.2	8.7 ​± ​0.1	8.0 ​± ​0.1	8.1 ​± ​0.1
IP3	10.5 ​± ​0.1	9.4 ​± ​0.2	9.3 ​± ​0.1	9.0 ​± ​0.1
MP4	5.8 ​± ​0.1	3.2 ​± ​0.1	3.0 ​± ​0.2	3.0 ​± ​0.1
MP5	4.8 ​± ​0.2	3.6 ​± ​0.1	3.3 ​± ​0.1	3.2 ​± ​0.1
CP6	9.9 ​± ​0.1	7.1 ​± ​0.1	7.1 ​± ​0.3	7.0 ​± ​0.1
CP7	10.7 ​± ​0.2	9.4 ​± ​0.1	8.3 ​± ​0.1	8.6 ​± ​0.1
CP8	6.5 ​± ​0.2	4.4 ​± ​0.2	3.3 ​± ​0.1	2.9 ​± ​0.1
CP9	7.3 ​± ​0.1	5.8 ​± ​0.2	5.8 ​± ​0.2	5.8 ​± ​0.1

*Bifidobacterium* sp.				
CP7	10.5 ​± ​0.2	9.2 ​± ​0.2	7.3 ​± ​0.1	7.2 ​± ​0.1
CP8	7.7 ​± ​0.1	3.3 ​± ​0.1	3.6 ​± ​0.1	3.5 ​± ​0.1
PC9	5.6 ​± ​0.2	5.4 ​± ​0.1	5.2 ​± ​0.1	5.1 ​± ​0.2
FM10	8.0 ​± ​0.1	6.7 ​± ​0.2	6.9 ​± ​0.1	6.9 ​± ​0.2
MP11	4.3 ​± ​0.1	3.5 ​± ​0.1	3.3 ​± ​0.2	3.3 ​± ​0.1

a Triplicate means followed by the standard deviation. Results expressed in log CFU g^−1^.

The regulation for the release of the commercialization of probiotic foods and supplements establishes that the manufacturer is responsible for proving to the inspection agency that the product confers the probiotic benefits to the consumer, that is, that the concentration and probiotic viability are sufficient to provide such effect during shelf life and, consequently, after consumption by the consumer (Brazil, 2018a; 2018b; FAO/WHO, 2006). Therefore, it is not possible to determine what would be the ideal concentration of probiotics in the product before consuming it.

However, studies show that for the food or probiotic supplement to offer such benefits, it must reach the ileum portion of the small intestine in a minimum concentration of 6 log CFU g^−1^ (Nazzaro et al., 2009; Hill et al., 2014). Still, it is expected that there will be a decrease of around 2 log CFU g^−1^ in the concentration of viable strains along the passage through the gastrointestinal tract, therefore, it is recommended that the food or supplement has in its concentration at least 8 log CFU g^−1^ (Vasiljevic and Shah, 2008).

Table 2 presents the results of the probiotic viability of the analyzed products, showing the initial concentration and survival during the simulation of the passage through the gastrointestinal tract. During the gastrointestinal simulation, it is possible to observe that there was a decrease in the concentration of viable cells in all 11 samples. Throughout the passage through the stomach, the viable cell count and the survival rate of probiotic microorganisms are reduced due to the extreme pH of stomach acid (Prestes et al., 2021; Cook et al., 2012), which limits the performance of probiotic microorganisms in the digestive system (Shori, 2017).

When analyzing the viability of the probiotic strains, it was possible to identify a decrease between 1 log CFU g^−1^ to approximately 4 log CFU g^−1^ during the passage through the gastrointestinal tract. Shindea et al. (2019) analyzing the functional efficacy of probiotics to simulated gastric conditions found a decrease in viability at the end of the gastric phase, with a drop in the cell count of 1.03 log CFU/mL at the end of the intestinal phase.

The samples for industrial purposes (IP1, IP2, and IP3), showed good initial concentration, above 8 log CFU g^−1^, after activation, and also after the simulated passage through the gastrointestinal tract, with an average decrease of 1.4 log CFU g^−1^, considered as expected.

The samples sold in handling pharmacies showed an initial concentration below 5 log CFU g^−1^, for both *Lactobacillus* and *Bifidobacterium.* After the simulation, the viability in the ileum portion of the intestine was around 3 log CFU g^−1^, considered low and probably this amount is insufficient to check the benefits of probiotics.

The CP6 sample consists of *Lactobacillus acidophilus* LA-14 surrounded by a capsule, with an initial concentration of 9.9 log CFU g^−1^, also presented on its label. After the simulated passage through the gastrointestinal tract, it presented a concentration of 7 log CFU g^−1^, an amount considered adequate to colonize the intestine.

The CP7 sample is composed of *Lactobacillus* and *Bifidobacterium,* also surrounded by a capsule, this sample showed an initial concentration of 10.7 log CFU g^−1^ for *Lactobacillus* and 10.5 log CFU g^−1^ for *Bifidobacterium*, corroborating with its packaging. After the in vitro test, the viability of 8.6 log CFU g^−1^ for *Lactobacillus* and 7.2 log CFU g^−1^ for *Bifidobacterium* was found, even with the reduction in the count, it is possible to offer the benefits to consumers.

The CP8 and CP9 samples, symbiotic, were sold in lyophilized form in sachets. The CP8 sample showed an initial concentration of 6.5 log CFU g^−1^ for *Lactobacillus* and 7.7 log CFU g^−1^ for *Bifidobacterium*, in disagreement with what was exposed in its labeling. After the in vitro test, it showed a decrease close to 4 log CFU g^−1^, considered low viability, which probably will not confer the probiotic benefits.

The CP9 sample also showed lower values than indicated on the label for *Lactobacillus* and *Bifidobacterium,* and after the gastrointestinal simulation, there was a decrease of around 1 log CFU g^−1^, considered more stable during digestion. The labeling for this sample indicates that daily consumption should be 1 to 2 sachets. If the individual chooses to consume 2 sachets per day, he will be receiving a concentration above 6 log CFU g^−1^, an amount that can already be considered sufficient to colonize the host's intestine.

The FM10 sample has no indication of concentration on its label, only consumption, which must be 200 ​mL/day. The initial viability found was 8 log CFU g^−1^, and after the in vitro test, there was a loss of 1 log CFU g^−1^, being within the recommended to check the probiotic benefits. Prestes et al. (2021) evaluated three probiotic fermented milk samples and found similar results, where the fermented milk maintained high viability (8–9 log CFU g^−1^) throughout the gastrointestinal tract, especially at the time of consumption.

Fermented milk presented results considered satisfactory, and it was the only probiotic analyzed commercialized in the food class and supermarkets. One explanation for the survival and good concentration of microorganisms in this product is that fat globules can have a protective effect on probiotic cells (Veruck et al., 2017). An advantage in relation to other samples, however, it requires low temperature during the storage period, even so, it is considered an effective matrix for probiotic stability (Prestes et al., 2021).

Numerous health benefits have been attributed to the ingestion of probiotics, however, it is important to emphasize that such benefits only occur if they are viable in the products where they will be included, that is, they survive during their processing and storage conditions, being thus ingested in adequate quantities, reaching a viable number of microorganisms (Sohail et al., 2011; Tripathi and Giri, 2014; Rouxinol-Dias et al., 2016).

Samples that have gone through the activation process are considered adequate, but the consumer will not be able to perform this process at home, which makes it impossible to indicate the consumption of these samples to the general public. The manipulated samples do not indicate to the consumer, not even the manufacturer's laboratory. Still, they can be considered a risk to the consumer, for not offering the benefits to the individual who needs it and also for leaving the administration at the consumer's choice.

The capsules involving the CP6 and CP7 samples show that they are efficient to maintain the probiotic concentration during the storage of the products, however, they cannot protect them from the action of gastric acids during digestion, as there was an average decrease of 2.7 log CFU g^−1^ during the gastrointestinal simulation. Even so, CP6 and CP7 samples showed the best results in these studies, and the preference for the consumption of probiotics coated by capsules can be indicated.

The symbiotic samples indicate that the presence of prebiotics does not increase its viability during storage and does not protect the microorganism during digestion, however, it can contribute to increasing colonization in the intestine, a test that was not performed in the present study. Still, it is important to note that the amount ingested daily is extremely important that the manufacturer's recommendation is followed so that the ideal concentration is offered to the host.

Dairy products, as they naturally contain probiotic microorganisms, tend to be a good source to keep them alive during storage.Verruck et al. (2017) explain that dairy derivatives contribute to the survival of probiotics in the gastrointestinal tract due to fat globules, which can protect viable cells against the extreme acidic conditions of the stomach and bile salts of the intestine. Also, it is a great possibility to offer probiotics and increases the chances of consumption by the population because it is being sold on supermarket shelves and not being seen as a medicine.

The most unsatisfactory results were seen in probiotics acquired in handling pharmacies, which are still sold without any indication of preparation and consumption. These probiotics, because they are not sold in the original packaging of the manufacturer, but in the packaging of the pharmacy that sells them, do not have a date of manufacture, only an expiration date, offering superficial information to the consumer.

It is known that exposure to oxygen and reduction in pH decreases the concentration of probiotics, as well as the presence of sucrose, has a potential inhibitor (Vinderola et al., 2002), still some products of the initial metabolism of lactic acid (diacetyl, acetaldehyde, acid lactic acid) may also be associated with loss of viability of probiotic bacteria (Prestes et al., 2021).

The low acidity of the stomach is the first barrier against microorganisms, many ingested bacteria die, however, acid resistant bacteria like Lactobacillus sp., Bifidobacterium sp. and Streptococcus sp. are able to survive (Guyton and Hall, 2002). However, bacteria with probiotic potential have a low acidification capacity and should be used in association with bio-adjusting cultures. Although pH is not the only factor involved in the growth of probiotic bacteria, its difficulty in maintaining viable cells at a pH below 4.0 reduces its concentration under these conditions (Carvalho Lima et al., 2009).

The contact time of the microorganism at low pH is a decisive factor for the viability of the cells. Slower digestions in the stomach stage will have less probiotic concentration. Also, the pH of the stomach varies throughout the day according to the food eaten, and it may be interesting to consume probiotics with foods and drinks that have a higher pH.

The probiotic samples analyzed that presented a low concentration of viable cells, were, in general, lyophilized products stored at room temperature and that need to be reconstituted in an aqueous medium. In this context, milk and some derivatives can protect microorganisms, helping to maintain viability during digestion when exposed to conditions with low pH (below 2.0), associated with this protection the presence of fat globules and milk proteins, mainly casein (Kos et al., 2000).

Thus, probiotic supplements could be more viable if they were associated with bacteria resistant to the acidic environment, serving these as protective cultures. Also, the consumption of these supplements associated with milk and some derivatives, such as fermented milk, may contribute to maintain or even increase the viability of probiotic cells during digestion.

For the moment, it is not possible to define the exact reasons that lead to decreased viability of probiotic cells in in vitro experimentation during digestion in the present study, since only the survival of microorganisms against the action of enzymes and pH changes was analyzed, with the addition of HCl and NaHCO3, which is possible to affirm that there is a decrease in the viable cell count.

The simulated method employed does not consider the physiological characteristics of the stomach and intestine, nor the other physical-chemical processes that occur during digestion in living beings. Becoming of great importance to carry out future tests with the intention of determining such effects together with probiotic viability under the conditions studied.

## 4 Conclusion

Only two samples had the same concentration as indicated on the product label. All samples showed a reduction in the concentration of probiotic microorganisms after the gastrointestinal simulation and only six showed a concentration in the ileum of the small intestine above 6 log CFU g^−1^, an amount estimated as the ideal to promote the probiotic benefits in the host.

More information is needed on the labeling of products, especially those marketed in handling pharmacies. It is necessary to increase the inspection on their production and commercialization. Still, the probiotics evaluated show the need to implement technologies to maintain the probiotic viability of microorganisms, so that they can meet the needs of consumers, as well as offer quality products that meet the expected benefits.

## Declaration of competing interest

The authors declare that they have no known competing financial interests or personal relationships that could have appeared to influence the work reported in this paper.
